# Variations in Soil Nutrient Dynamics and Bacterial Communities After the Conversion of Forests to Long-Term Tea Monoculture Systems

**DOI:** 10.3389/fmicb.2022.896530

**Published:** 2022-06-24

**Authors:** Heng Gui, Lichao Fan, Donghui Wang, Peng Yan, Xin Li, Yinghua Pang, Liping Zhang, Kazem Zamanian, Lingling Shi, Jianchu Xu, Wenyan Han

**Affiliations:** ^1^Tea Research Institute, Chinese Academy of Agricultural Sciences, Hangzhou, China; ^2^Department of Economic Plants and Biotechnology, Yunnan Key Laboratory for Wild Plant Resources, Kunming Institute of Botany, Chinese Academy of Sciences, Kunming, China; ^3^Centre for Mountain Futures (CMF), Kunming Institute of Botany, Chinese Academy of Sciences, Kunming, China; ^4^Department of Soil Science of Temperate Ecosystems, University of Göttingen, Göttingen, Germany; ^5^Yunnan Key Laboratory for Fungal Diversity and Green Development, Kunming Institute of Botany, Chinese Academy of Sciences, Kunming, China; ^6^Bureau of Agriculture and Rural Affairs of the Yuhang District, Hangzhou, China; ^7^School of Geographical Sciences, Nanjing University of Information Science and Technology, Nanjing, China

**Keywords:** tea production, pyrosequencing, monoculture system, co-occurrence network, nutrient availability

## Abstract

The soil microbial community is a key indicator to evaluate the soil health and productivities in agricultural ecosystems. Monoculture and conversions of forests to tea plantations have been widely applied in tea plantation globally, but long-term monoculture of tea plantation could lead to soil degradation and yield decline. Understanding how long-term monoculture systems influence the soil health and ecosystem functions in tea plantation is of great importance for soil environment management. In this study, through the comparison of three independent tea plantations across eastern China composed of varying stand ages (from 3 to 90 years after conversion from forest), we found that long-term tea monoculture led to significant increases in soil total organic carbon (TOC) and microbial nitrogen (MBN). Additionally, the structure, function, and co-occurrence network of soil bacterial communities were investigated by pyrosequencing 16S rRNA genes. The pyrosequencing analysis revealed that the structures and functions of soil bacterial communities were significantly affected by different stand ages, but sampling sites and land-use conversion (from forest to tea plantation) had stronger effects than stand age on the diversity and structure of soil bacterial communities. Soil bacterial diversity can be improved with increasing stand ages in tea plantation. Further RDA analysis revealed that the C and N availability improvement in tea plantation soils led to the variation of structure and function in soil bacterial communities. Moreover, co-occurrence network analysis of soil bacterial communities also demonstrated that interactions among soil bacteria taxa were strengthened with increasing stand age. Our findings suggest that long-term monoculture with proper managements could be beneficial to soil ecosystems by increasing the C and N content and strengthening bacterial associations in tea plantations. Overall, this study provides a comprehensive understanding of the impact of land-use change and long-term monoculture stand age on soil environments in tea plantation.

## Introduction

Soil microbial communities play an indispensable role in maintaining soil health and nutrient cycling (Torsvik et al., 1996; Bünemann et al., 2018), and are strongly affected by environmental factors such as soil pH, soil texture, and nutrient availability (Girvan et al., 2003; Lauber et al., 2009; Geisseler and Scow, 2014), plant species (El Zahar Haichar et al., 2008), land management (Rodrigues et al., 2013), and location (Fierer and Jackson, 2006; Martiny et al., 2006; Decaëns, 2010). Among these factors, land-use change is the most impactful factor through which humans disturb soil environmental conditions, thereby altering the structure, diversity, and biomass of bacterial communities (Da C Jesus et al., 2009). Recent advances in high-throughput sequencing approaches now enable us to understand that the type of vegetation planted (El Zahar Haichar et al., 2008) and management (Geisseler and Scow, 2014) along a chronosequence (i.e., land-use duration) after land-use conversion play important roles in controlling the variation of soil bacterial communities through reshaping the soil structure and affecting the accumulation of soil nutrients and hazardous substances. Microorganisms usually form complex interactive networks in which interactions among members are essential for community assembly and ecosystem functions (Deng et al., 2016; Shi et al., 2016). Therefore, identifying and defining the interactions that occur among soil microorganisms are critical to understanding microbial diversity and functions (Banerjee et al., 2016). Network analysis of co-occurrence, which is usually determined by correlations between abundances of microbial taxa, provides a promising start for exploring the organization and dynamics of microbial interactions and niches (Barberán et al., 2012; Jiao et al., 2016; Chen et al., 2019). Exploring these microbial interactions, rather than those of simple microbial richness and composition in the soil environment, can provide important information on plant health and growth, especially in agriculture soils (Banerjee et al., 2016; Zhang et al., 2018). For example, high-throughput sequencing approaches allow researchers to define network complexity or stability between microbial communities and environmental factors (Ma et al., 2020), or to identify potential keystone species (Fan et al., 2018) or geographical patterns at a continental scale (Zhang et al., 2018). However, there is little information about topological variation found in soil microbial co-occurrence interactions in long-term monoculture agricultural systems.

Tea (*Camellia sinensis* L.) is a perennial evergreen broad-leaved cash crop (Han et al., 2007), and tea is one of the top three most widely consumed beverages in the world. Tea plantations are typically established by conversion from forest through years (decades to centuries) of intensively managed cultivation practices (e.g., agriculture tillage, heavy pruning; Han et al., 2007). Most tea plantations are distributed in subtropical areas, and over 3.06 million ha are established in China, a figure that continues to increase (Fan and Han, 2020). Soil degradation is one potential issue arising from the long-term monoculture of tea plantations (Yan et al., 2018, 2020) due to the accumulation of C and N caused by the litterfall from the heavy pruning and annual application of the organic and N fertilizers (Fan et al., 2015; Fan and Han, 2020). To better understand the mechanisms undergirding the soil nutrient-cycling networks in tea plantations, which promote soil fertility as well as the production and quality of tea, it is of vital importance to document the influence of land-use change and long-term monoculture on soil microbiomes in tea plantations. Accordingly, increased attention has been concentrated on soil microbiomes in tea plantations, such as the study of the microbial community structure and microbial biomass as affected by the stand age of tea plantations (Wang et al., 2019). However, the relative importance of long-term monoculture systems and spatial variation on the structure and function of bacterial communities in tea plantations remains unclear.

**Figure 1:**
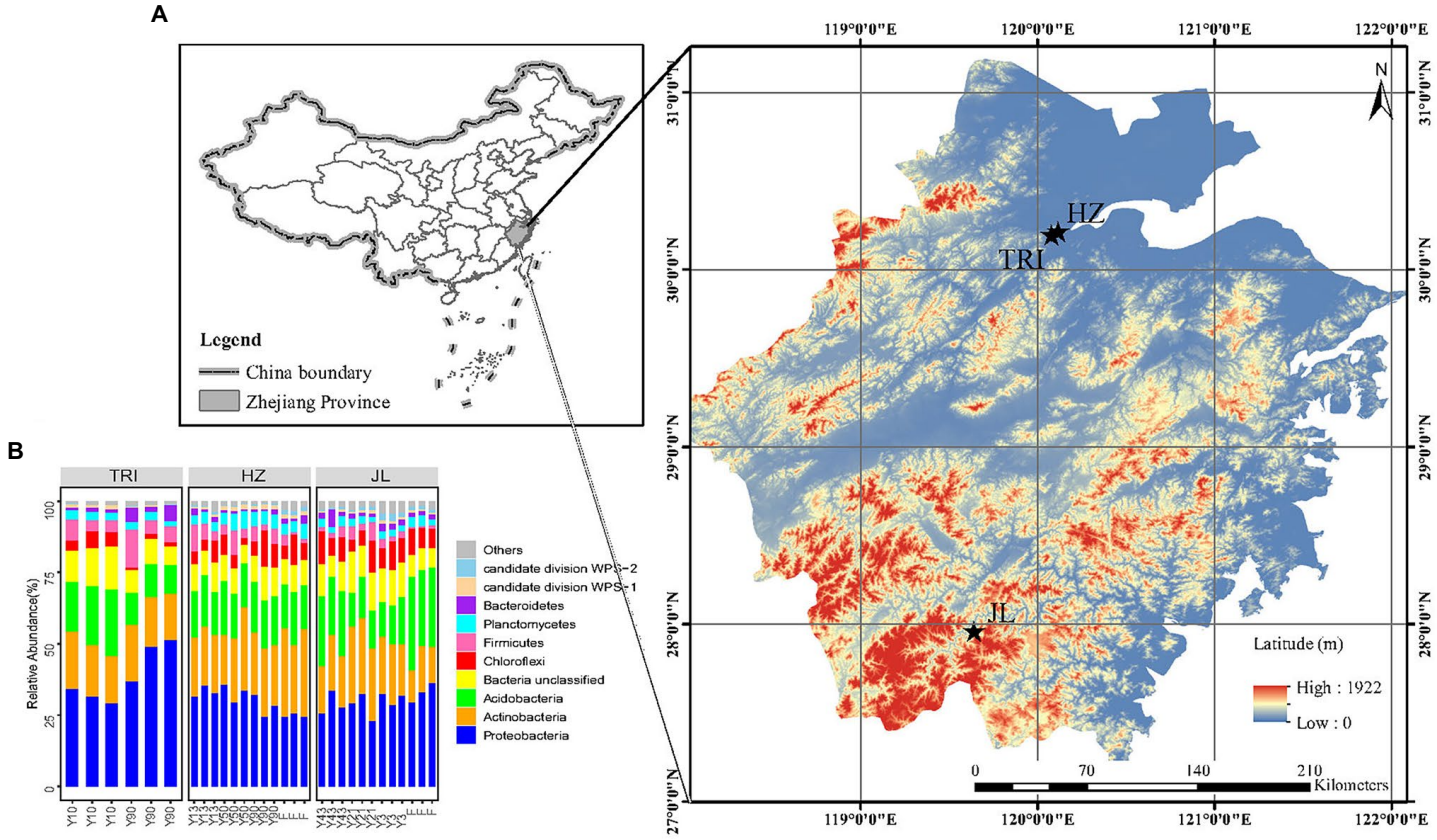
**(A)** Sampling sites of tea plantations in Zhejiang Province, China (TRI: the Tea Research Institute of the Chinese Academy of Agricultural Sciences; HZ: Wenjiashan village, Hangzhou city; JL: Jingning county, Lishui city). **(B)** Taxonomic structure of the soil bacterial microbiota at the phylum level. Only the 10 phyla with the highest mean relative abundance are shown, while the other phylum groups are grouped into “others.”

Here, we analyzed soil bacterial communities *via* pyrosequencing analysis at varying stand ages (from 3 to 90 years) across tea plantations at three separate sites (Figure 1A) and their adjacent forests. Our study aimed to answer the following research questions:

Q1. How do stand age (time after land conversion) and land-use change affect soil properties and the structure and function of the soil bacterial communities in tea plantations?
Q2. Which possible environmental factors especially soil properties could lead to the changes in soil bacterial communities described in Q1?
Q3. How does stand age affect the interactions in soil bacterial communities in tea plantations based on co-occurrence network analysis?

## Materials and Methods

### Experimental Design and Soil Sampling

The sampling of tea plantation sites is shown in Figure 1A, and selected environmental information is provided in Table 1. In general, three tea plantations composed of tea stands with varying stand ages in Zhejiang Province, China, were chosen for comparing changes in soil bacterial communities between different tea stand ages. The first plantation was located in Jingning County, Lishui City (JL), in which three tea stands aged three (Y3), 21 (Y21), and 43 (Y43) years were selected. The second was located at the Tea Research Institute of the Chinese Academy of Agricultural Sciences (TRI), in which two tea stands aged 10 (Y10) and 90 (Y90) years were selected. The last plantation was located in Wenjiashan Village, Hangzhou City (HZ), in which three tea stands aged 13 (Y13), 50 (Y50), and 90 (Y90) years were selected. For these three different sites, the annual mean temperature is 17°C, ranging from 1.7°C in January to 33.0°C in July. The annual mean precipitation is 1,533 mm, with 74% of total rainfall occurring during the tea growing season from March to September. At the JL and HZ sites, one forest (the land use prior to clearing and planting tea, and no tea plantation history) adjacent to the tea stand was selected for comparison. As described in Han et al. (2007), evergreen latifoliate forest vegetation in both the HZ and JL sites was dominated by *Cyclobalanopsis glauca* and *Quercus acutissima* Carri. The management practices for different tea stands at each tea plantation were similar. As described in our previous study (Han et al., 2007), 2,250 kg ha^−1^ organic fertilizer (mainly rape seed cake) containing 45% organic C, 4.6% N, 0.9% P, and 1.2% K was applied for each tea stand in every September or October. For the adjacent forest, no fertilizer or any agricultural management was applied. Soil tillage and weeding was conducted together with the application of organic fertilizers in all tea plantations. The prunings were left *in situ* as surface mulch. No pesticides or bio-pesticides such as the nuclear polyhedrosis virus, *Bacillus thuringiensis* and matrine were used to control pests. Other agricultural managements like pruning and plucking, were the same as applied in all tea plantations.

**Table 1:** Selected information for the three tea plantations and soil physicochemical properties in tea plantations under different sites and stand ages.

Site	TRI		HZ				JL			
MAT (°C)	17		17				17.5			
MAP (mm)	1,533		1,533				1,599			
US Soil classification	Ultisols		Ultisols				Ultisols			
Stand age	10	90	F^#^	13	50	90	F^#^	3	21	43
Sample size (m^2^)	400	400	400	400	400	400	400	400	400	400
pH (H_2_O, 1:1)	3.95 ± 0.1b	3.93 ± 0.23b	4.32 ± 0.05ab	4.17 ± 0.04ab	3.9 ± 0.34b	3.92 ± 0.06b	4.35 ± 0.11ab	4.38 ± 0.31ab	3.95 ± 0.2b	4.56 ± 0.17a
TOC (g kg^−1^)	2.11 ± 0.01b	2.69 ± 0.05a	1.11 ± 0.09f	1.53 ± 0.31cd	1.64 ± 0.11c	1.95 ± 0.07b	1.05 ± 0.06f	1.06 ± 0.03f	1.25 ± 0.09ef	1.35 ± 0.08de
TON (g kg^−1^)	0.22 ± 0.03b	0.26 ± 0.01a	0.1 ± 0.01ef	0.12 ± 0.02cde	0.14 ± 0.01cd	0.15 ± 0.02c	0.08 ± 0.01f	0.11 ± 0.01def	0.12 ± 0.01cde	0.14 ± 0.01cde
C/N	9.9 ± 1.35b	10.46 ± 0.21b	11.07 ± 0.29ab	12.49 ± 0.59ab	11.71 ± 1.16ab	13.03 ± 1.7a	13.41 ± 1.53a	10.02 ± 0.53b	10.18 ± 0.63b	10.06 ± 1b
MBC (mg kg^−1^)	493.1 ± 51.77b	650.13 ± 21.49a	431.67 ± 33.75b	246.75 ± 54.75de	350.67 ± 33.47c	277.82 ± 38.64cd	86.13 ± 17.46f	66.68 ± 16.65f	175.42 ± 60.31e	597.72 ± 89.15a
MBN (mg kg^−1^)	15.38 ± 2.46bc	52.2 ± 9.06a	12.32 ± 0.67c	9.54 ± 2.52c	17.12 ± 1.46bc	13.85 ± 4.56c	11.76 ± 3.45c	11.63 ± 0.93c	24.23 ± 2.28b	15.89 ± 6.28bc
AP (mg kg^−1^)	45.25 ± 10.96ab	66.89 ± 44.18a	7.16 ± 3.28b	32.78 ± 2.97ab	37.57 ± 6.78ab	40.25 ± 11.9ab	3.17 ± 2.42b	1.73 ± 0.6b	22.11 ± 10.16b	4.51 ± 1.11b
Exch. K (mg kg^−1^)	71.73 ± 11.61ab	97.57 ± 16.72ab	109.63 ± 42.95a	87.88 ± 17.95ab	51.09 ± 4.96b	70.16 ± 18.35ab	61.77 ± 2.64ab	83.09 ± 11.07ab	67.67 ± 8.28ab	65.74 ± 12.08ab
Exch. Ca (mg kg^−1^)	575.63 ± 124.19ab	1,137 ± 1039.23ab	1531.5 ± 955.34a	706.37 ± 222.65ab	419.23 ± 46.02ab	294.77 ± 120.6b	191.57 ± 19.82b	341.67 ± 35.2b	266.7 ± 141.45b	320.03 ± 79.09b
Exch. Mg (mg kg^−1^)	39.88 ± 14.89a	72.29 ± 59.78a	31.65 ± 4.83a	29.54 ± 7.21a	21.29 ± 1.8a	19.72 ± 8.27a	20.06 ± 2.82a	39.01 ± 1.98a	25.95 ± 3.15a	40.26 ± 32.94a
Exch. Na (mg kg^−1^)	3.62 ± 0.96b	2.91 ± 0.7b	8.96 ± 0.77a	5.47 ± 0.81ab	6.9 ± 1.98ab	6.62 ± 1.42ab	4.45 ± 1.91b	6.99 ± 2.15ab	3.15 ± 1.55b	4 ± 2.05b

# Where forest is represented by “F.”

MAT, mean annual temperature and MAP, mean annual precipitation.

### Soil Sampling and Treatments

For each forest at HZ and JL sites and tea stand at each tea plantation, three 400 m^2^ plots of representative soil were randomly selected for soil collection. For each plot, eight random soil sub-samples (5 cm in diameter) at 0–20 cm depth were taken and mixed into one independent soil sample for later related analysis. Before soil sampling, the litter layer was removed at each plot. The soil was sieved using a 2-mm pore-size screen to remove plant roots, stones, and soil fauna. Each independent soil sample was divided into two parts. One portion (50 g) was stored at 4°C for later soil physicochemical analysis. The other portion (10 g) was stored at −80°C for later DNA extraction. All the soil samplings and analysis were conducted in 2013.

### Soil Analysis

Soil pH was determined by a combination of glass electrodes using a 1:2.5 (w:v) ratio of soil to distilled water. Soil total C (TOC) and N (TON) were measured by a LECO CNS Combustion Analyzer (LECO, CNS 2000, LECO Corporation, Michigan, United States) following the manufacturer’s protocol. Soil microbial biomass C (MBC) and N (MBN) were determined following the fumigation-extraction method. As described in our previous study (Han et al., 2007), briefly, the C concentration was determined using a Multi C/N 3100 TOC analyzer (Analytik Jena AG, Jena, Germany), and a value of kEC = 0.45 was used to calibrate biomass C content. Biomass ninhydrin-N concentrations were measured colorimetrically. Available phosphorus (AP) was extracted using hydrochloric acid and ammonium fluoride and determined using the molybdenum blue method. The concentrations of exchangeable K (Exch. K), Ca (Exch. Ca), Mg (Exch. Mg), and Na (Exch. Na) were tested following hot block acid digestion protocol (Huang and Schulte, 1985).

### Soil Microbial DNA Extraction and PCR Amplification

Total DNA was extracted from about 0.5 g of soil from each sample using the Mo Bio PowerSoil DNA isolation kit (Carlsbad, CA, United States) according to the manufacturer’s instructions. After extraction, the quality and concentration of DNA were tested using the NanoDrop ND 200 spectrophotometer (Thermo Scientific, United States). In accordance with the concentration, all DNA samples were diluted to 1 ng/uL before PCR amplification.

The V4 and V5 variable regions of the bacterial 16S rRNA gene were amplified using the primers 515F (5’-CCATCTCATCCCTGCGTGTCTCCGAC-3′) and 907R (5’-CCTATCCCCTGTGTGCCTTGGCAGTC-3′). The PCR amplification mixture was prepared with 1 μl purified DNA template (10 ng), 5 μl 10 × PCR buffer, 2.25 mmol L^−1^ MgCl_2_, 0.8 mmol L^−1^ deoxyribonucleotide triphosphate (dNTP), 0.5 μmol L^−1^ of each primer, 2.5 U Taq DNA polymerase, and sterile filtered Milli-Q water to a final volume of 50 μl. All reactions were carried out in a PTC-200 thermal cycler (MJ Research Co., New York, United States). PCR cycles included a 4 min initial denaturation at 94°C, followed by 30 cycles of denaturation at 94°C for 1 min, annealing at 53°C for 30 s, extension at 72°C for 1 min, and a 5-min final elongation step at 72°C. PCR products were quality-screened and purified using the Qiagen Gel Extraction kit (Qiagen, Hilden, Germany).

### 454 Pyrosequencing and Sequencing Processing

Pyrosequencing was performed on a Roche Genome Sequencer FLX+ using Titanium chemistry by Macrogen (Roche Applied Science, Mannheim, Germany). Three standard flow-gram format (SFF) files were generated by 454 pyrosequencing. The SFF file was analyzed by the software package mothur (version 1.33.2) following the protocol provided by https://mothur.org/wiki/454_SOP. Briefly, de-noising and chimera analysis conducted with the AmpliconNoise (Quince et al., 2011) and UCHIME algorithms were used to reduce sequence errors. Furthermore, quality trimming was conducted to remove unwanted sequences shorter than 200 bp and reads containing ambiguous bases and with homopolymers longer than eight bases. Sorting and trimming were performed using the Pipeline Initial Process at the RDP Pyrosequencing Pipeline with the default settings.^1^ Remaining sequences were used to identify unique sequences by aligning with the SILVA-based bacteria reference alignment (version 128). Within unique sequences, the Uchime tool was applied to remove chimeras. Next, “Chloroplast,” “Mitochondria,” or “unknown” was identified and removed from the dataset. Subsequently, after calculating the pairwise distance and generating the distance matrix, a 97% identity threshold was used to cluster sequences into operational taxonomic units (OTUs) according to the UCLUST algorithm (Edgar, 2010). For each OTU, the SILVA database was applied to annotate taxonomic information. To compensate for different sequencing depths, all the samples were rarefied to an even depth of 7,000 reads for 16S rRNA sequences before statistical analysis.

### Network Construction and Analysis

In order to determine the effects of different stand ages on microbiome associations in soils, underlying co-occurring bacterial taxa were depicted through co-occurrence network analysis. We divided all soil samples from all tea stands into four groups according to their land-use types and stand ages: (1) Forest (F); (2) 3–20-year-old stands (Y3–20); (3) 40–50-year-old stands (Y40–50); and (4) 90-year-old stands (Y90). In order to reduce the complexity of the network, Spearman’s correlation between two families was considered statistically robust if Spearman’s correlation coefficient (*r*) was >0.8 and the value of *p* was <0.01 (Barberán et al., 2012). Meanwhile, a multiple testing correction using the Benjamini–Hochberg (FDR) method was applied to adjust the values of *p* and reduce the chance of obtaining false-positive results (Benjamini and Hochberg, 1995). All robust correlations identified from pairwise comparison of family abundance formed a correlation network in which the node represented bacterial family taxa and the edge represented a strong and significant correlation between the nodes. In addition, we generated sub-networks for each soil sample from meta-community networks by preserving the OTUs presented in each tea stand with the subgraph function in igraph packages (Ma et al., 2016). To describe the complex pattern of interrelationship between bacterial taxa, a set of topological characteristics (number of nodes and edges, average path length, network diameter, average degree, graph density, clustering coefficient, and modularity) was determined using the psych (Revelle, 2017), vegan (Oksanen et al., 2013), and igraph (Csardi and Nepusz, 2006) packages in the R environment (version no.: 3.60). Networks were visualized using the interactive platform Gephi (Bastian et al., 2009). In addition, 10,000 Erdős–Rényi (ER) random networks were generated to compare the topology of the real network with a random graph, which connected each pair of nodes with any probability (Erdős and Rényi, 1960).

### Statistical Analysis

Maps of sampling sites were generated using GenGIS, All the other statistical analyses were performed in the R environment (version no.: 3.60). Soil physicochemical properties were ordinated by principal component analysis (PCA) using the R package *FactoMineR* v1.0.7 (Lê et al., 2008). To compare differences in the soil physicochemical properties and alpha diversity of soil bacterial communities between different stand ages, a repeated measures ANOVA followed by multiple pairwise comparison using Tukey’s test at *α* = 0.05 was conducted using the Rpackage *ggpubr* v0.30. Linear discriminant analysis effect size (LEfSe) was performed to elaborate potential bacterial taxa (from phylum to genus) within soil microbiomes that specifically enrich different stand ages of tea plantations, based on *p* < 0.05 and a LDA score > 2.0 (Segata et al., 2011). The alpha-diversity (richness) of soil bacterial communities was estimated based on OTUs. To assess changes in soil bacterial community structures (beta-diversity) among different tea management practices and stand ages, principal coordinate analysis (PCoA) was used to calculate the gradient in compositional changes of bacterial microbial communities (based on Bray-Curtis distances). The alpha- and beta- diversity were calculated using the R package *microeco* v0.90 (Louca et al., 2016; Liu et al., 2021). Permutation multivariate analysis of variance (PERMANOVA) was employed to assess the effects of stand ages and sites on the soil bacterial community using the *Adonis* function of the R package *vegan* v2.6-1 (Bell et al., 2014). Distance-based redundancy analysis (db-RDA) was conducted to identify soil physicochemical properties with a significant impact on soil bacterial communities across different stand ages of tea plantations using the *dbrda* function of the R *vegan* package vegan v2.6-1. Parameters that significantly explained variation in the bacterial community were identified using forward selection (the *ordistep* function of the *vegan* package) with *p* value <0.05. FAPROTAX (Functional Annotation of Prokaryotic Taxa) was applied to predict the microbial ecological function profiles using the *trans_func* class of the R package microeco v0.90 (Louca et al., 2016; Liu et al., 2021). Non-metric multidimensional scaling (NMDS) analysis based on Bray-Curtis distances was used to evaluate the compositional changes in microbial function groups between different sites and stand ages. Spearman’s correlation analysis between the Euclidean distances of standardized sub-network topological parameters was applied to explore the effects of soil properties on sub-network topological features. Soil properties (except for pH) were normalized before the correlation analysis. Pearson’s correlation analysis was applied to investigate the relationship between individual sub-network topological parameters (average path length, centralization betweenness, No. of edges and No. of vertices) and stand age of tea plantation. The network parameters were ln-transformed before the correlation analysis. All the correlation analysis were performed using R package *psych* v2.0.8 (Revelle, 2020). All the data visualization were completed using R package *ggplot2* v3.3.2.

## Results

### Soil Properties

**Figure 2:**
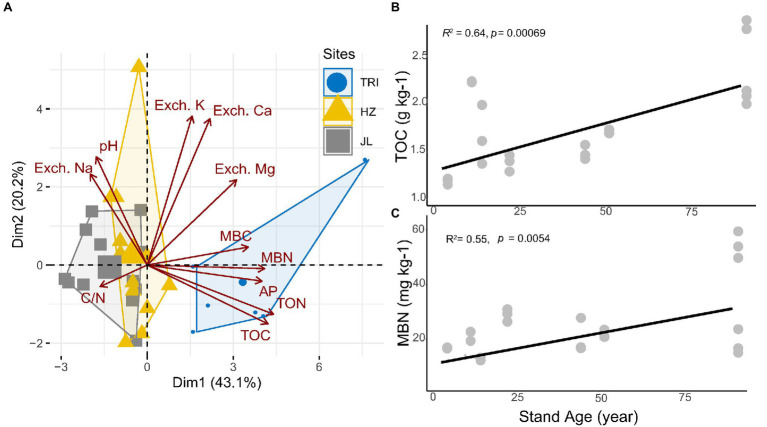
**(A)** Principal component analysis (PCA) based on soil physicochemical properties as variables. The sampled tea stands from same site are outlined and grouped as the same color. **(B,C)** show significant linear regression (*p* < 0.01) between total carbon (TOC) and microbial biological nitrogen (MBN), respectively, in the soil and the stand age across all tea plantations.

Soil pH and 10 other soil properties (TOC, TON, C/N, MBC, MBN, AP, Exch. K, Exch. Ca, Exch. Mg, and Exch. Na) are listed in Table 1. Based on detected soil properties, PCA showed that soil samples from the TRI site featured significant environmental heterogeneities across different stand ages (Figure 2A). The content of TOC and TON increased along with the stand ages across the three sampling sites (Table 1), and the correlation analysis indicated that as the stand age of tea plantations increased, TOC and MBN in soil increased significantly (*p* < 0.01; Figures 2B,C). However, no consecutive changes along with increased stand age were recorded for other soil properties (including MBC, AP, and Exch. K; Table 1).

### The Bacterial Component at Different Phylogenic Levels

Sequencing the amplicon libraries resulted in a total of 341,915 raw reads prior to quality checking and assigning reads to their respective sample. The average read length (±standard deviation) of reads before processing was 405 ± 96 bp. After quality trimming and assigning reads to different samples, 204,723 high quality reads remained in the dataset with an average length of 207 ± 4 bp. The number of high-quality sequences per sample varied from 7,265 sequences to 9,204 sequences (mean = 8,530).

The dominant bacterial phyla across all samples were Proteobacteria, Actinobacteria, Acidobacteria, Chloroflexi, and Firmicutes, while on average 15% of the reads could not be classified (Figure 1B). To detect soil bacteria taxa that were significantly affected by tea stand age and location, the LEfSe analysis based on OTUs was applied to compare the differences. In general, the change trend of bacterial taxa varied between sites. LEfSe analysis revealed that 16 taxa affiliated with three phyla (e.g., the families of *Porphyromonadaceae*, *Enterococcaceae*, *Lactobacillales*, *Alcaligenaceae*, *Burkholderiales*, *Neisseriaceae*, *Xanthomonadaceae* and *Acholeplasmataceae*) increased significantly (*p* < 0.05, LDA > 2.0) in the 90-year-old tea plantation, while 13 biomarkers within four phyla (e.g., the family of *Geodermatophilaceae*, *Intrasporangiaceae*, *Streptomycetaceae*, *Peptostreptococcaceae*, *Beijerinckiaceae*, *Methylobacteriaceae* and *Xanthomonadaceae*) decreased significantly in the 10-year-old tea plantation in TRI. Moreover, LEfSe analysis demonstrated that few bacterial taxa increased with years of tea planting in JL (e.g., the family of Brucellaceae and Rhizobiaceae) and HZ sites (e.g., the family of *Xanthobacteraceae*) compared to the forest soil (F; Supplementary Figures S1, S2).

### Bacterial Community Diversity and Structure

**Figure 3:**
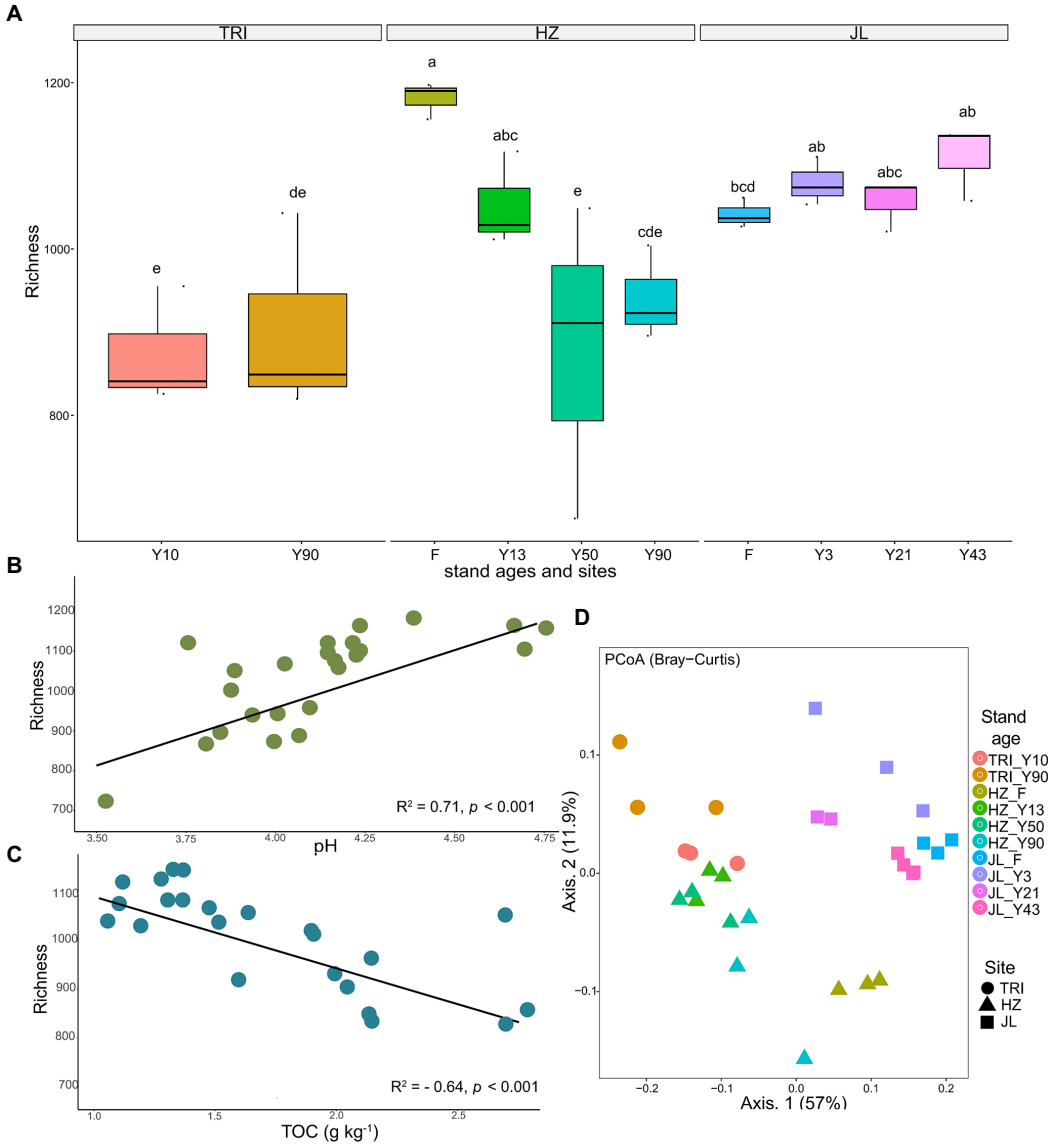
**(A)** The richness of the tea plantation soil bacterial communities at varying stand ages and different sampling sites. **(B,C)** show significant linear regression (*p* < 0.001) relationships between total carbon (TOC) and soil pH in the soil, respectively, and the richness of soil bacterial communities across all tea stands. **(D)** Principal coordinate analysis (based on Bray–Curtis distances) of soil bacterial community composition across varying stand ages and different sampling sites. The samples are separated by sites (TRI, HZ, and JL; represented by different shapes) and stand ages [F (adjacent forest); represented by different colors].

The bacterial richness, or alpha diversity, varied widely across different sites (Figure 3A). Soil bacterial richness did not change significantly at different stand ages across all sites, except at the HZ site, which saw a significantly higher richness in F compared to that in tea plantations. Further Spearman’s correlation analysis revealed that soil bacterial richness was significantly positively correlated with pH (*R*^2^ = 0.71, *p* < 0.001) and negatively correlated with TOC (*R*^2^ = −0.53, *p* < 0.001) in tea plantations (Figures 3B,C, respectively).

To explore how changes in microbiome structure and composition correlated with sampling sites and stand age, we computed the between-sample diversity (β-diversity) using Bray-Curtis distance. Axis 1 and axis 2 explained 57% and 11.9% of the total variation in bacterial community structure, respectively. The principal coordinate analysis (PCoA) of the bacteria community structure revealed that soil samples from different sites with different stand ages were generally clustered separately (Figure 3D).

### Soil Bacterial Functions

**Figure 4:**
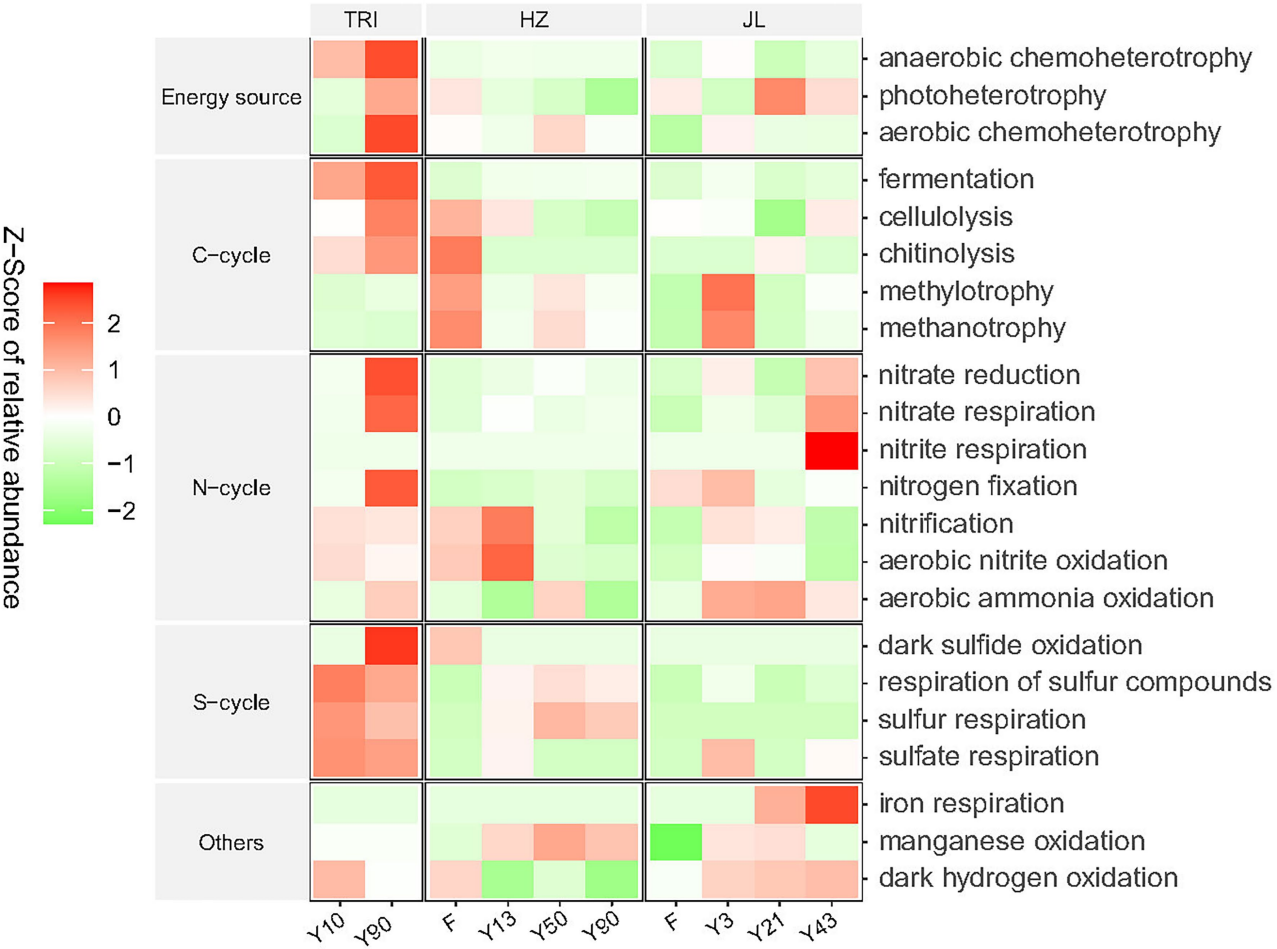
Function predictions of microbial communities in tea plantation soils across varying stand ages and different sampling sites by FAPROTAX. The relative abundance of each functional category is normalized and represented by a Z-score.

A total of 22 functional sub-categories (relative abundance > 1%) within five major categories, “Energy source,” “C-cycle,” “N-cycle,” “S-cycle,” and “Others,” were identified and linked to the microbial communities across different forest, tea stand ages and sites (Figure 4). No consecutive change of these functional sub-categories was observed along with the increasing tea stand years in all three sites. Nevertheless, the results in Figure 4 showed that in the TRI site, tea plantation soils with greater stand ages had a higher relative abundance of chemoheterotrophy, photoheterotrophy, fermentation, cellulolysis, chitinolysis, nitrate reduction, nitrate respiration, nitrogen fixation, and aerobic ammonia oxidation. In addition, in the JL site, the relative abundance of dark hydrogen oxidation increased with the stand ages. When converting forest to tea plantation, in the HZ site, the relative abundance of most C-cycle functions decreased. The non-metric multidimensional scaling (NMDS, based on Bray-Curtis distance) plot of all 22 sub-categories showed separate clusters of functional categories between TRI and the soils of the other two sites (Supplementary Figure S3A). In the TRI and JL sites, the clusters from different tea stand soils were separated from each other, suggesting that there were significant differences in the function of soil microbial communities between different tea stand ages (Supplementary Figure S3B).

### Co-occurrence Network Analysis of Soil Bacterial Communities

**Figure 5:**
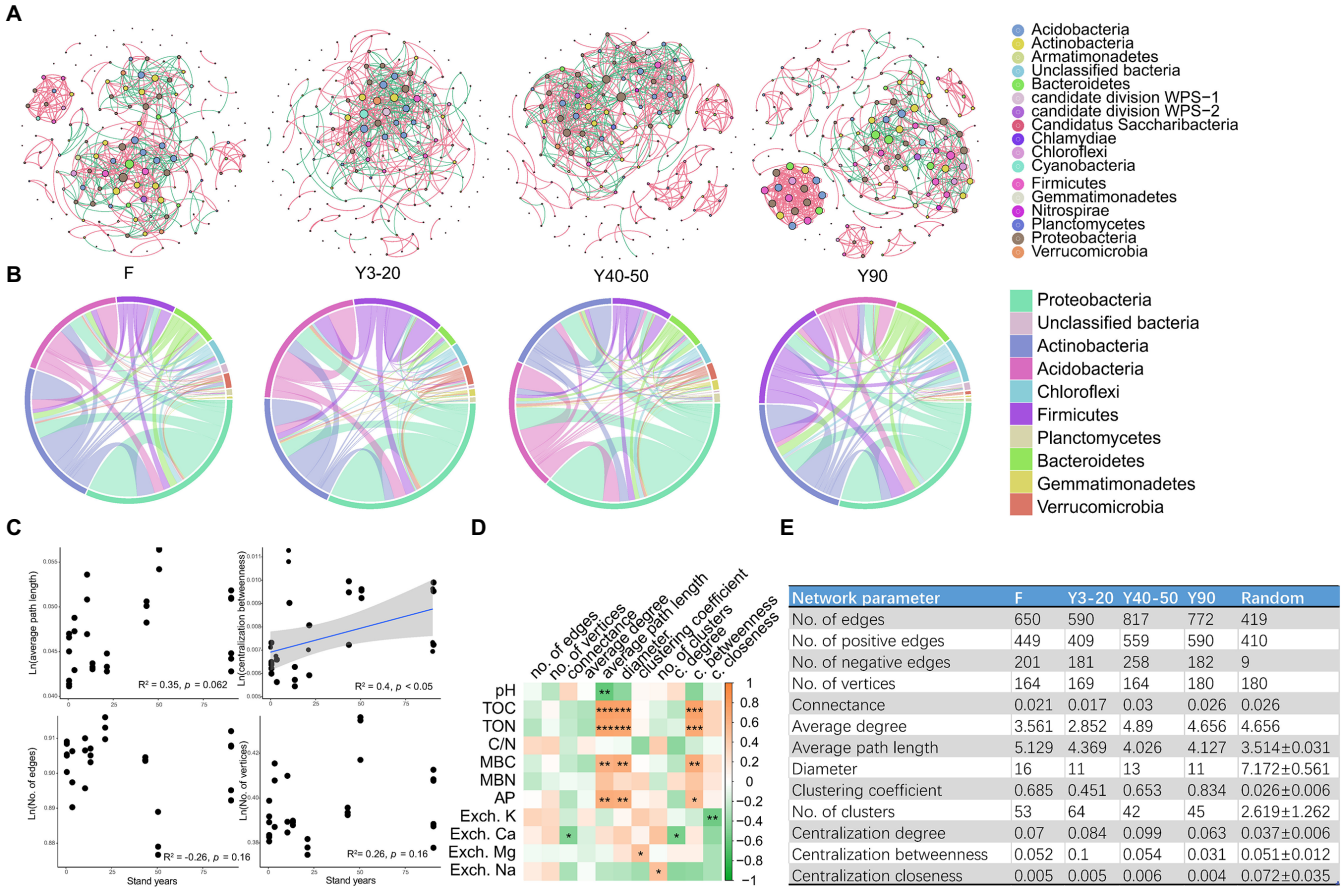
**(A)** The co-occurrence networks visualize the effects of varying stand ages of tea plantations (Y3–20, Y40–50, and Y90) and the adjacent forest (F) on the co-occurrence pattern between soil bacterial taxa at family level. The node size is proportional to the abundance of taxa, and the nodes represent bacterial taxa at the family level. The edges are colored according to interaction types; positive correlations are labeled with green, and negative correlations are colored in pink. **(B)** CIRCOS plots showing the distribution of links among the top 10 interacting phyla in networks Y3–20, Y40–50, Y90, and F. **(C)** Linear regression relationships between tea stand ages and key topological parameters (ln-transformed) of all subnetworks (average path length, centralization betweenness, no. of edges, and no. of vertices). **(D)** Spearman’s correlations between soil physicochemical properties and topological parameters in all subnetworks. Significant correlations are marked by *p < 0.05, **p < 0.01, and ***p < 0.001. **(E)** Topological parameters of the networks Y3–20, Y40–50, Y90, and F.

For co-occurrence network analysis, we divided all samples into four groups: (1) Forest (F); (2) 10–20-year-old tea stands (Y10–20); (3) 40–50-year-old tea stands (Y40–50); and (4) 90-year-old tea stands (Y90). Subsequently, four networks (F, Y3–20, Y40–50, and Y90) were constructed to test the effect of stand age on the soil bacterial communities’ association. Overall, nodes in all networks were assigned to 15 bacteria phyla and three unclassified groups. Co-occurrence networks were markedly different among different stand ages (Figure 5A). Most links were derived from the phyla of Proteobacteria, Actinobacteria, Acidobacteria, Firmicutes, and Bacteroidetes across the four networks we constructed (Figure 5B). However, the proportion of each phyla varied with different networks. In the F network, three phyla (Proteobacteria, Actinobacteria, and Acidobacteria) accounted for over 75% of the total links, but this proportion decreased in other tea stand networks (Figure 5B). We then investigated the correlations between key topological parameters of the subnetworks and stand ages using Pearson’s correlation analysis. The centralization betweenness positively and significantly correlated with stand age (*p* < 0.05), suggesting that the importance of individual bacterial community groups became more uniform as stand age increased. However, the average path length (APL), the number of edges and vertices showed no significant correlation with stand ages (Figure 5C). Further Spearman’s correlation analysis between topological parameters and soil physicochemical properties revealed that soil C, N, and P were all significantly correlated with some key parameters of subnetworks (such as APL, diameter, and centralization betweenness; Figure 5D). When comparing the network parameters we calculated in Figure 5E between the four co-occurrence networks, the results showed that the number of positive edges was much higher than that of the negative edges across the soils from all stand ages as well as that of forest soil. Furthermore, values relating to the APL, clustering coefficient, and numbers of clusters in those empirical networks of various tea plantations and forests were higher than those of their respective, identically sized Erdős–Rényi random networks. This indicated that the empirical networks had significant “small-world” modularity and hierarchy of their topological properties (Figure 5E). Further structural analysis showed that the clustering coefficient and edge numbers of the networks increased along with stand ages (Figure 5E), indicating that the increased tea plantation stand age caused soil bacterial community associations to become tighter and more complex. In addition, compared with F soil, all tea plantation soil bacterial communities had a relatively high APL value (Figure 5E).

### Relationship Between Soil Properties and the Structure, Function, and Co-occurrence Pattern of Soil Bacterial Communities

**Figure 6:**
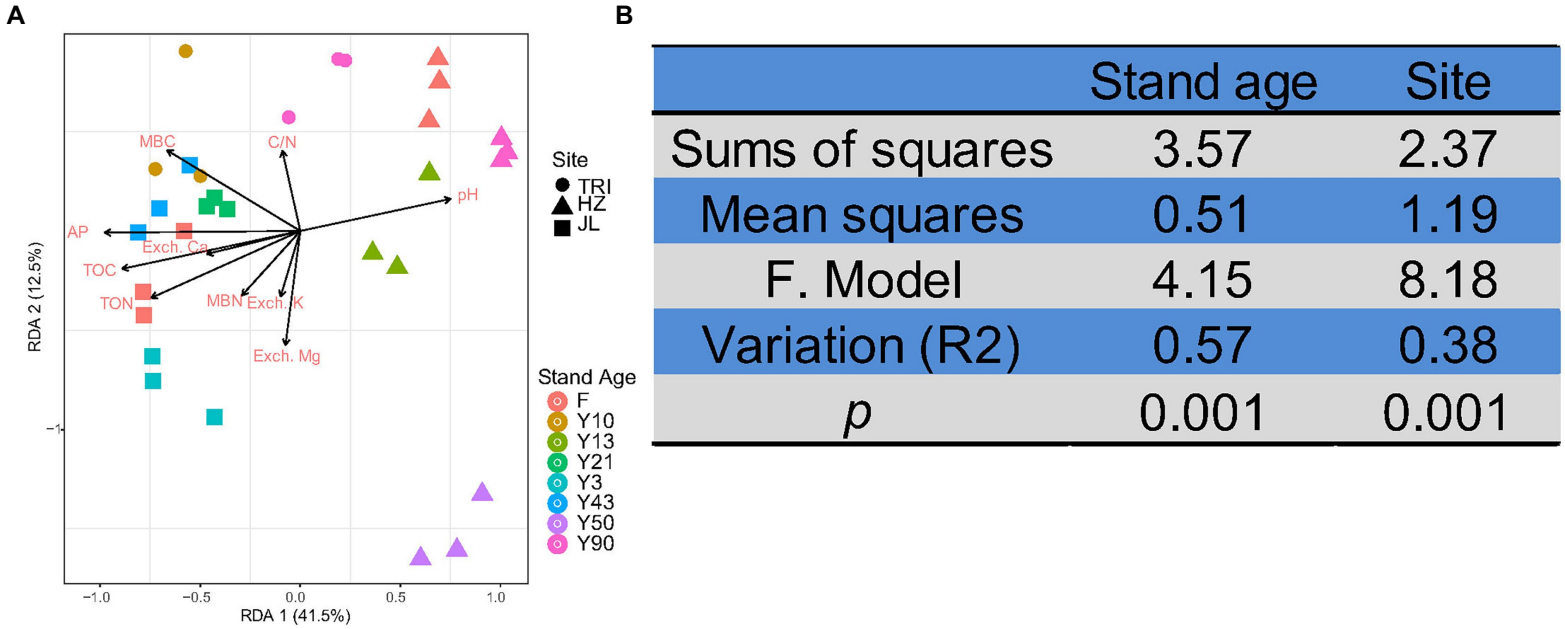
**(A)** Distance-based redundancy analysis (RDA) of the relationships between soil physicochemical properties and bacterial communities at different sites and stand ages. The samples are separated by sites (TRI, HZ, and JL; represented by different shapes) and stand ages [including the adjacent forest (F); represented by different colors]. **(B)** The effects of stand age and sampling site on soil bacterial communities of tea plantations based on permutation multivariate analysis of variance (PERMANOVA).

Db-RDA was applied to study the effects of soil properties on the structure of soil bacterial communities based on OTU abundance. The ordination diagram showed that bacterial community change was significantly correlated with the soil variables TOC, TON, MBC, pH, and AP&K (*p* < 0.05, Monte Carlo test; Figure 6A). The first two axes of RDA explained 41.5% and 12.5% of the total variation. In addition, both sampling sites and stand age significantly affected soil bacterial communities (PERMANOVA test, *p* < 0.01), and stand age (*R*^2^ = 0.569) outcompeted sampling sites (*R*^2^ = 0.377; Figure 6B) for controlling bacterial community composition.

## Discussion

Soil microbial communities are of particular relevance in tea cultivation, since soil microbiota represent reservoirs of microorganisms colonizing tea plantations and contribute to improved yield and tea quality (Mortimer et al., 2015). To characterize the effects of long-term monoculture and other environmental factors like location and soil properties on soil bacterial communities, we investigated the soil bacterial communities of tea plantations at different stand ages as well as adjacent forests at three different sites in eastern China.

### Effect of Soil Properties on Shaping Soil Bacterial Communities

Our study revealed that soil TOC and MBN in tea plantations are significantly positively correlated with stand age (Figures 2B,C). This is in line with previous studies reporting the plantation age as a critical factor affecting SOC and N dynamics during land-use change, in particular on tea plantations (Pansombat et al., 1997; Wang et al., 2016). This demonstrates that long-term tea plantations result in a significant accumulation of organic C and N. The increase in the amount of soil micro-aggregates in long-term tea plantations may be a reason for such an increase in TOC and MBN, as micro-aggregates are the most predominant pools of SOC and other nutrients (Wang et al., 2016). In addition, as described in our previous study (Gui et al., 2021), the application of long-term organic or mineral fertilizers in tea plantations could also result in the accumulation of organic C and N in the soil. Regular agricultural managements including pruning and tillage could also affected nutrient input and the accumulation of C and N in tea gardens soils (Syswerda et al., 2012). The shift of these key nutrients in soil with different stand age might have resulted in the changes of soil bacterial community. For instance, in TRI site, the abundance of Gammaproteobacteria were increased due to the long-term tea monoculture, which may be explained by that Gammaproteobacteria are usually associated with most available nutrients (Li et al., 2016). Pseudomonadaceae and Xanthomonadales are believed to be abundant in suppressive soil than in conducive soil and related with plant growth (Mendes et al., 2011). LEfSe analysis in TRI and JL sites showed the abundance of these two bacterial groups decreased with increasing stand ages.

Soil pH is an important factor affecting soil bacterial communities at regional and global scales, which has been confirmed in other ecosystems (Delgado-Baquerizo et al., 2018). For instance, Zhou et al. (2017) found that the richness of soil bacteria was significantly negatively correlated with soil pH in rubber plantations (pH: 3.94–4.41), but in an oil-contaminated soil, pH was positively associated with bacterial diversity (pH: 7.49–9.20; Jiao et al., 2016). Our research has shown that in an acidic tea plantation, soil pH was strongly positively correlated with the alpha diversity of soil bacterial communities (Figure 3B). This finding is in line with previous studies (Griffiths et al., 2011), which have illustrated that soil pH is positively correlated with bacterial alpha diversity, and that the alpha diversity of bacterial communities is the highest at a near-neutral pH. After surveying soils from a wide array of ecosystem types in America continent, Fierer and Jackson (2006) proposed that soil pH is an independent driver of soil bacterial diversity and richness, and bacterial diversity was highest in neutral soils and lower in acidic soils. Our research has shown and confirmed the similar trend of soil bacterial diversity in tea garden soils. It has been proved that any significant deviation of environmental (extracellular) pH can cause stress on single-celled microorganisms, as the intracellular pH of most microorganisms tend to be neutral (Madigan et al., 1997; Fierer and Jackson, 2006). Therefore, it is reasonable that environmental pH including soil pH can be a controlling effect on the overall diversity and composition of soil microbial communities in various ecosystems (Jiao et al., 2016; Amoakwah et al., 2022; Nan et al., 2022).

In addition to the importance of some commonly accepted variables like C, N, and soil pH in shaping soil microbial communities, our study also supports the finding that soil base cations like Ca and K are important in shaping the composition and co-occurrence network of tea plantation bacterial communities (Figure 6). These base cations act as nutrients or structural components of the cells of living microorganisms (Tripler et al., 2006). When considering the drivers of soil bacterial communities, the availability of Ca and K mostly impacts the bacteria involved in the dissolution of soil minerals (e.g., mineral-weathering bacteria; Puente et al., 2004). The effects of Ca and K on the structure of soil bacterial communities have been reported in forest soils (Uroz et al., 2011) and agricultural soils (Schmidt et al., 2019). Our study reaffirmed the importance of these base cations in shaping bacterial community structures and intra-taxa associations in tea plantation soils.

### Effects of Tea Plantation Stand Age on Microbial Communities

The alpha diversity of soil bacterial communities remained stable in long-term tea plantations (Figure 3A). This sort of stability was also observed in a 20-year-old tea plantation (Li et al., 2016). Zhao et al. (2012) proposed that in monoculture cropping systems, such as tea plantations, rhizosphere effects are the critical factors that determine the bacterial community diversity and the toxicity and accumulation of antimicrobial substances due to long-term cropping, as well as the specific acidic soil environment, which may suppress the development of bacterial populations. One or a combination of these factors could explain the stable alpha diversity observed in our study. Furthermore, soil properties like pH are known to intimately determine bacterial diversity and community composition (Bissett et al., 2011). As shown in Table 1, soil pH did not show a significant response to long-term tea plantations at each site, which could also have resulted in the insignificant change of the alpha diversity. In addition, despite the acidic soil environment, the increasing TOC and MBN with successive years of tea planting may also contribute to the stability of the alpha diversity, as soil organic matter and nutrients have a profound effect on microbial diversity (Montecchia et al., 2015). Previous studies (Jangid et al., 2008; Lee-Cruz et al., 2013) have shown that the alpha diversity of soil bacterial communities can be decreased by the conversion of forests to long-term agriculture management. In contrast, our finding at the JL site did not agree with this conclusion. This suggests that after forests are converted to a monoculture agricultural system, it does not necessarily mean that the bacterial community’s diversity is reduced or lost. Despite that the managements for tea plantations in JL site were the same as other sites, there are two reasons to explain the different response in JL site. Firstly, unlike the adjacent forest in HZ site, the forest in JL site has relatively lower MBC content compared with tea plantations and with increasing tea stand age, the content of MBC increased gradually (Table 1). Higher content of MBC in the soil usually represents higher alpha-diversity of soil microbial communities (Zheng et al., 2019). Secondly, we think the different response of JL site is due to the geographical distance or origin. As shown in Figure 1A, JL site locates far from the other two sites (TRI and HZ), which has different geographical origin and elevation. Our study also revealed that some key soil functions related to C and N cycles shifted after the forest was converted to tea plantation through PROTAXA prediction (Figure 4A). This disagreement is mostly because the effect of cultivation on alpha diversity and soil functions strongly depends on the nature of the soil and the cultivation type (Coller et al., 2019). Since the quality of SOM was already low at pH 4.3 in the forest areas, a 0.4 unit decrease in pH in long-time plantations did not make a considerable difference in SOM quality. Therefore, the microbial response remained comparable between tea plantations and forests. In addition, the uncertainly of the function prediction could also lead to the disagreement, since the PROTAXA used in our study is based on DNA metabarcoding data and using taxonomic gene information and is lack of reference genomes especially for complex ecosystems such as soils (Djemiel et al., 2022).

Considering the between-samples variability, both PERMANOVA and PCoA analyses indicated that sampling location and stand age significantly affected the beta diversity of soil bacterial communities (*p* < 0.01), and the effect of stand age (*R*^2^ = 0.569) was more significant than that of the sampling location (*R*^2^ = 0.377; Figure 6B). It has been previously suggested that geographical origin and soil type variation were the dominant factors in determining the structure of soil bacterial communities in vineyards (Coller et al., 2019), which is partially consistent with our finding in tea plantation soils. Importantly, we found that stand age could indeed shape the structure of bacterial communities in tea plantation soil. Because this change in microbial community structure was mostly induced by C and N increases with increasing tea stand age, the bacterial community structure is mainly affected by environmental variables (Jiao et al., 2016). In general, our study confirmed that environmental variability caused by long-term monoculture and spatial variability (tea plantation sites) determined the structure of bacterial communities in tea plantation soil.

### Interactions Among Soil Bacteria Taxa Were Strengthened by Long-Term Tea Monoculture

Referring to the studied bacterial communities, the 16S rRNA sequencing indicated that Proteobacteria, Actinobacteria, and Acidobacteria were the dominant taxa across all samples in tea plantation soils. This finding is consistent with previous Chinese tea plantation soil studies (Li et al., 2016). A co-occurrence network analysis across all stand ages also revealed that most of the nodes belonged to these three phyla. In addition, we found that the tea plantation networks were nonrandom and typically matched the topological features of a small-world and intrinsic modular architecture (Barberán et al., 2012). This typical “small-world” characteristic in tea plantation soils made the networks stronger than random associations (Watts and Strogatz, 1998).

Interestingly, to our knowledge, this study is among the first reporting that long-term tea monoculture tightened soil microbial associations. One possible explanation is that changes in some taxa were sensitive to C and N increases induced by tea planting. The LEfSe analysis detected several carbon- or nitrogen-susceptible taxa in the Rhizobiales, Xanthomonadales, and Burkholderiales, which have previously been reported as keystone taxa in agricultural ecosystems linked to C and N metabolism in soils (Li et al., 2015). In our study, the function prediction also revealed that some bacterial taxa, including Actinobacteria and Chthonomonadetes, were correlated with the key processes in C and N cycles (Figure 4B). Another explanation is that greater nutrient availability, including increased C and N, in the soil could subsequently strengthen microbial interactions in order to enhance the efficiency of resource turnover, which benefits tea growth (Shi et al., 2016; Zhao et al., 2019). Lastly, according to the topological characteristic analysis, long-term tea monoculture reduced betweenness centralization and the links between key bacterial taxa in the networks, which could partially be caused by the tightening soil bacterial associations in tea plantations with increasing stand ages (Kuzyakov and Zamanian, 2019).

In order to display the chronosequence development of networks with stand ages, we merged tea stands soils from different sampling sites into one stand age group. While we acknowledge that this merge could obscure the effects of geographical parameters on network construction, this study is the first to demonstrate that long-term monoculture tightened soil microbiome network associations in tea plantations.

## Conclusion

Through comparison of three independent sites, we found that long-term tea monoculture led to significant increases in TOC and MBN. This C and N availability improvement in tea plantation soils associated with increasing tea stand age could contribute to tea yield growth and be of greater benefit to tea monocultural systems. The analysis of the 16S rRNA genes of bacterial communities revealed that the structures of soil bacterial communities were significantly altered by the stand age of tea plantations, sampling locations, and land-use conversion. Stand age and associated soil properties change like C and N availability improvement had a greater effect than sampling locations on the composition of bacterial communities. Interestingly, this study is the first of its kind to report that long-term tea monoculture tightened soil microbiome associations through co-occurrence network analysis.

## Data Availability Statement

The datasets presented in this study can be found in online repositories. The names of the repository/repositories and accession number(s) can be found at: https://www.ncbi.nlm.nih.gov/, PRJNA679995.

## Author Contributions

HG: investigation, software, and writing—original draft preparation. LF: data curation and writing—reviewing. DW: visualization and formal analysis. PY and YP: project administration. XL and LZ: software and visualization. KZ: writing—reviewing and editing. LS: reviewing and editing. JX: funding acquisition. WH: project administration, funding acquisition, and supervision. All authors contributed to the article and approved the submitted version.

## Funding

This research was supported by the National Key R&D Program of China (2017YFE0107500) and the National Natural Science Foundation of China (NSFC grant no. 32001296). KZ would like to thank the support from the Research Fund for International Young Scientists of National Natural Science Foundation of China (grant no. 42050410320). HG would also like to thank the support from the Youth Innovation Promotion Association of CAS, China (grant no. 2022396).

## Conflict of Interest

The authors declare that the research was conducted in the absence of any commercial or financial relationships that could be construed as a potential conflict of interest.

## Publisher’s Note

All claims expressed in this article are solely those of the authors and do not necessarily represent those of their affiliated organizations, or those of the publisher, the editors and the reviewers. Any product that may be evaluated in this article, or claim that may be made by its manufacturer, is not guaranteed or endorsed by the publisher.

## Footnotes

^1^ http://rdp.cme.msu.edu/
