# Supplementary material for: Variations in Soil Nutrient Dynamics and Bacterial Communities After the Conversion of Forests to Long-Term Tea Monoculture Systems
Source: Front Microbiol. 2022 Jun 24;13:896530. doi: 10.3389/fmicb.2022.896530 (PMC9263701; doi:10.3389/fmicb.2022.896530)
Supplement: Supplementary file 1 [file Data_Sheet_1.DOCX]

**Variations in soil nutrient dynamics and bacterial communities after the conversion of forests to long-term tea monoculture systems**

Heng Gui^1,2,3^, Lichao Fan^1,4,5*^, Donghui Wang^1^, Peng Yan^1^, Xin Li^1^, Yinghua Pang^6^, Liping Zhang^1^, Kazem Zamanian^4,7^, Lingling Shi^2,3^, Jianchu Xu^2,3^, Wenyan Han^1*^

^1^ Tea Research Institute, Chinese Academy of Agricultural Sciences, Hangzhou 310008, China

^2^ Department of Economic Plants and Biotechnology, Yunnan Key Laboratory for Wild Plant Resources, Kunming Institute of Botany, Chinese Academy of Sciences, Kunming 650201, China

^3^ Centre for Mountain Futures (CMF), Kunming Institute of Botany, Chinese Academy of Sciences, Kunming 650201, China

^4^ Department of Soil Science of Temperate Ecosystems, University of Göttingen, Göttingen 37077, Germany

^5^ Yunnan Key Laboratory for Fungal Diversity and Green Development, Kunming Institute of Botany, Chinese Academy of Sciences, Kunming, Yunnan, China

^6^ Bureau of Agriculture and Rural Affairs of the Yuhang District, Hangzhou, 310008, China

^7^ School of Geographical Sciences, Nanjing University of Information Science and Technology, Ningliu Road 219, Nanjing 210044, China

Corresponding Authors: Lichao Fan, [flcxsy@126.com](mailto:flcxsy@126.com), Wenyan Han [hanwy@tricaas.com](mailto:hanwy@tricaas.com)

**Supplementary Fig.** Sampling sites of tea plantations in Zhejiang Province, China (TRI: the Tea Research Institute of the Chinese Academy of Agricultural Sciences; HZ: Wenjiashan village, Hangzhou city; JL: Jingning county, Lishui city).


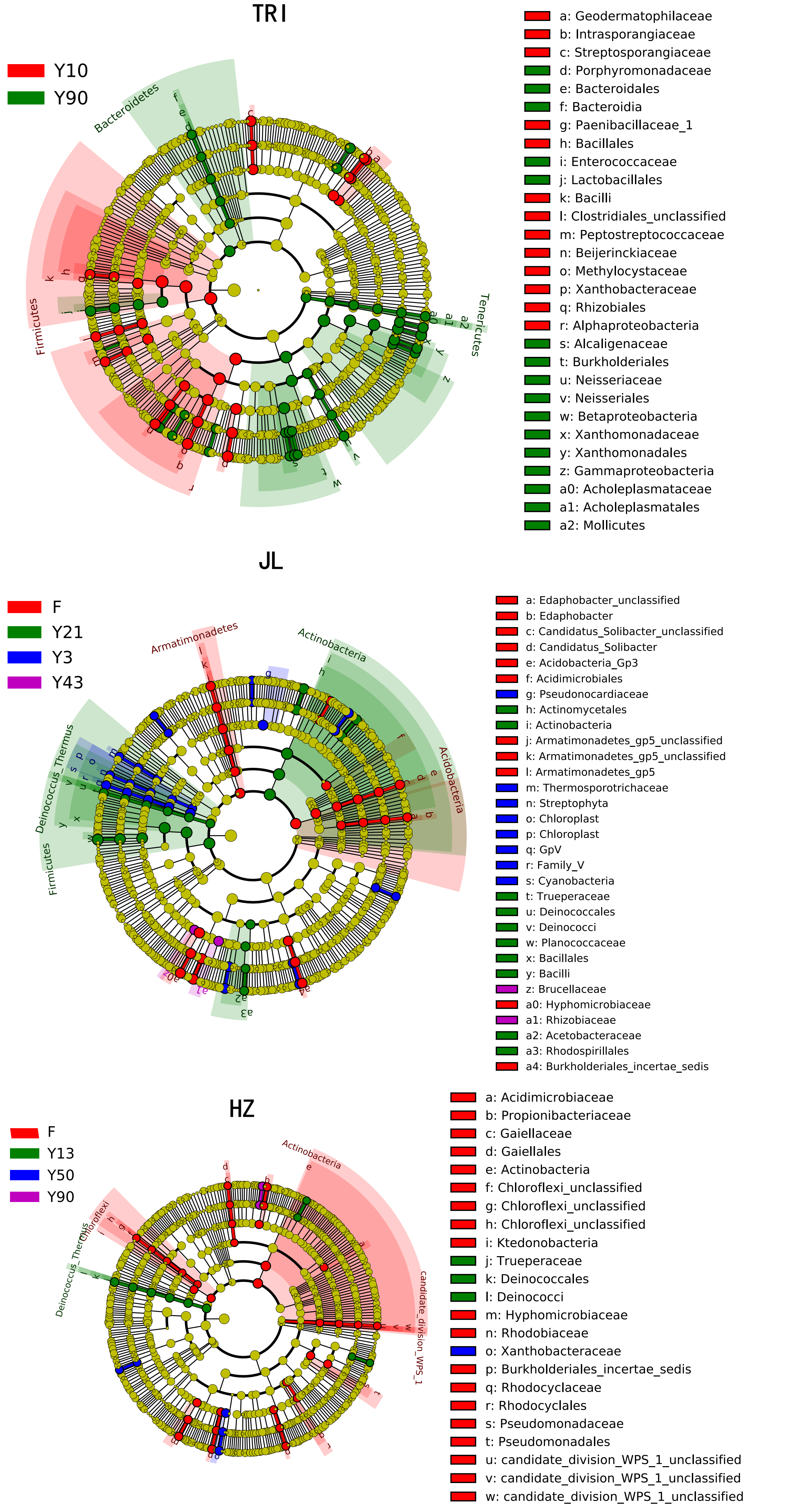


**Supplementary Fig. S1.** The effects of varying stand ages on the relative abundance of soil bacterial lineages were assessed through LDA Effect Size (LEfSe) with an absolute logarithmic LDA score threshold of 2.0 at three sampling sites (TRI, HZ, and JL). There are six circular rings in the cladogram, and each circular ring includes all taxa within a taxonomic level. From the inside to the outside, the circular rings represent supergroup, phylum, class, order, family, and genus.


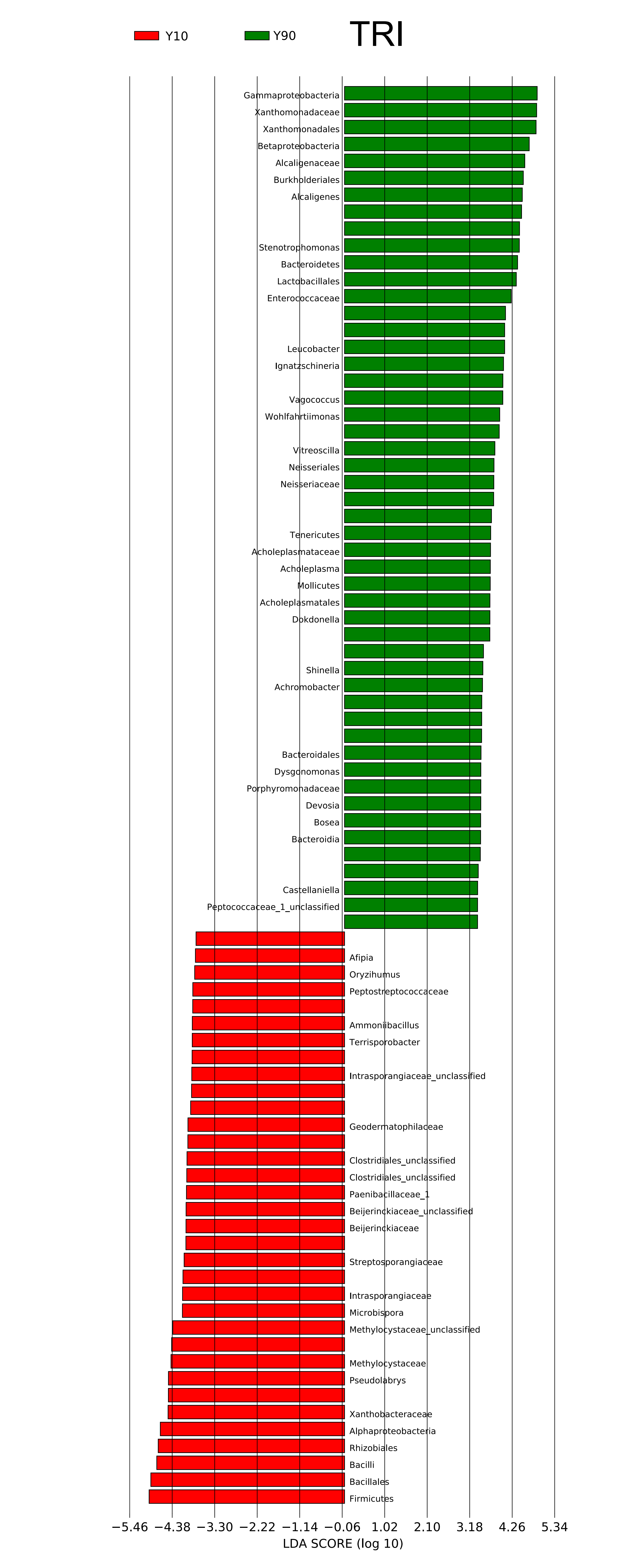


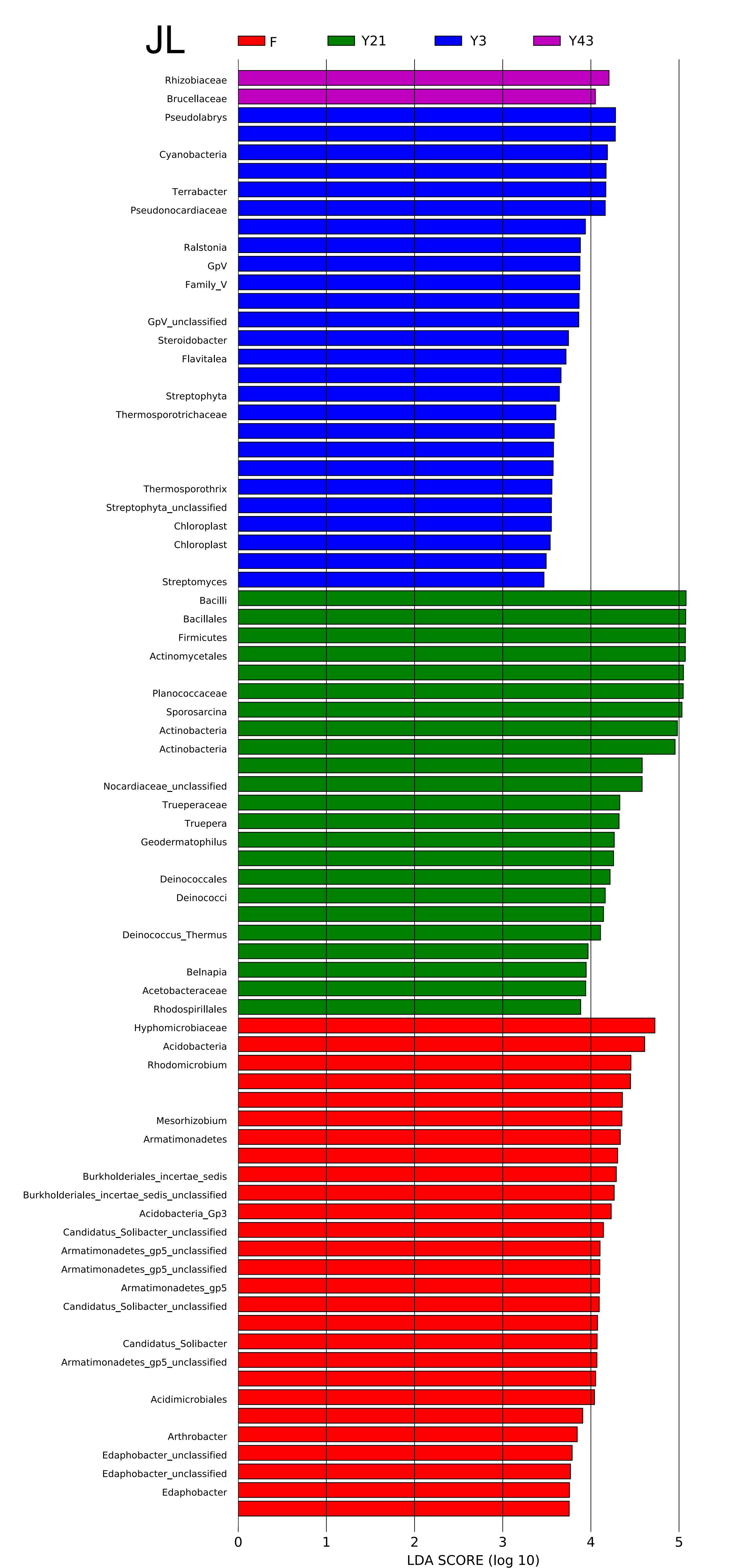


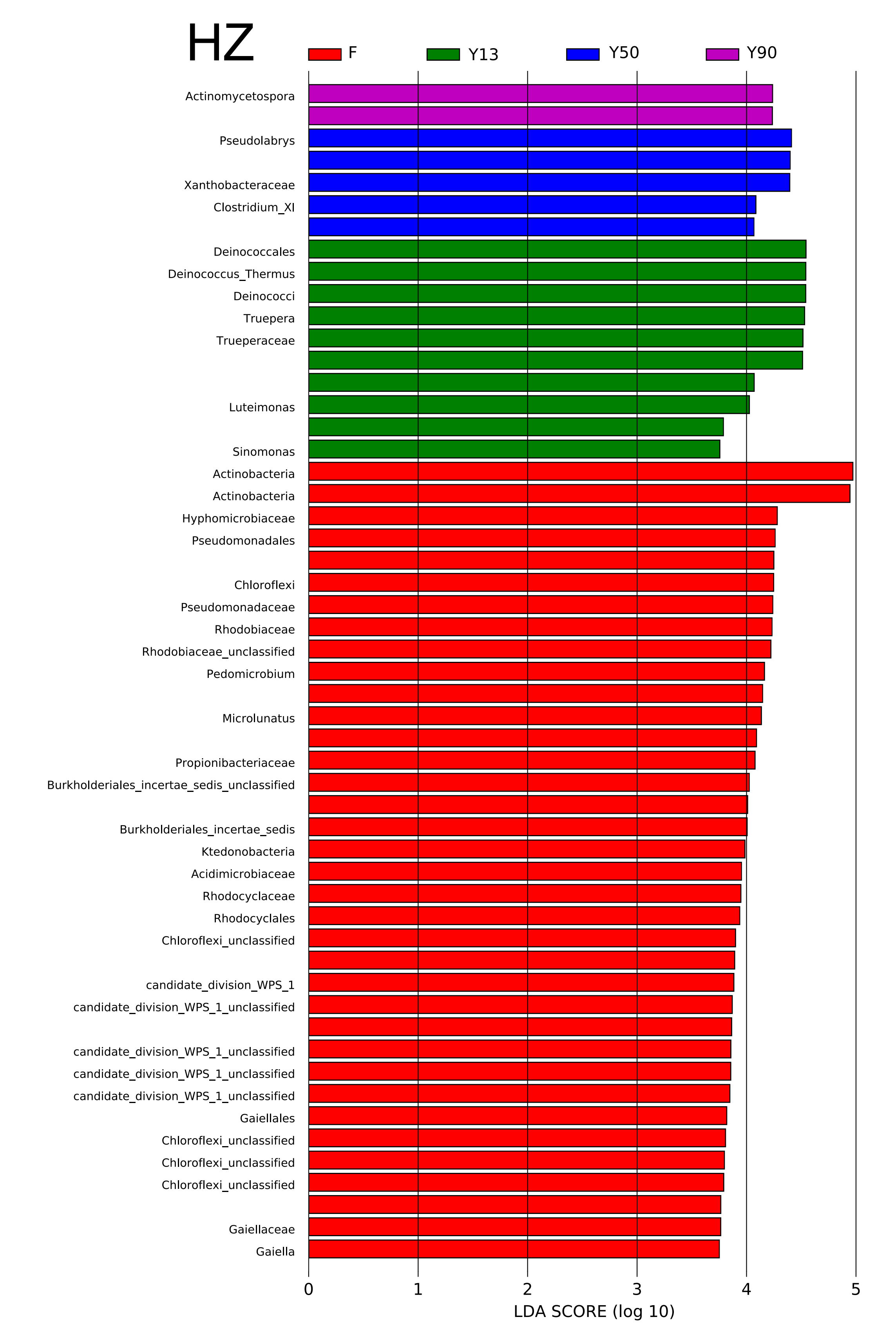


**Supplementary Fig. S2.** LDA Effect Size analysis showing the significant different microbial taxa in TRI, HZ and JL sites.


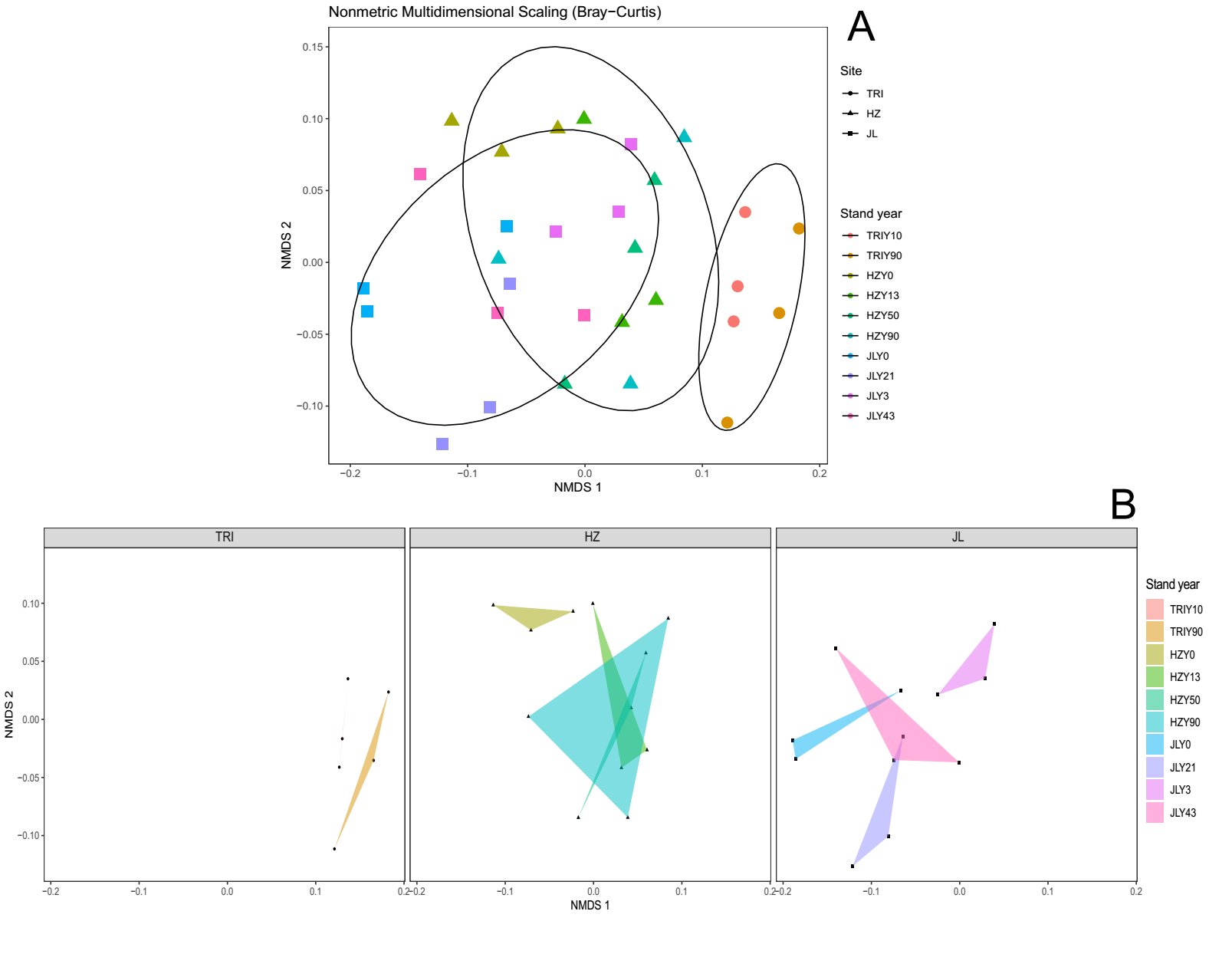


**Supplementary Fig. S3** Non-metric multidimensional scaling (NMDS) ordination based on Bray-Curtis distances under functional categories predicted by FAPROTAX (Stress value = 0.188). (A) and (B) show the NMDS ordination of all 3 sites and 3 each site separately. The samples separated by sites (TRI, HZ, and JL, respectively; represented by different shape) and stand ages (F (adjacent forest); represented by different colors).
